# INteractive Virtual Expert-Led Skills Training: A Multi-Modal Curriculum for Medical Trainees

**DOI:** 10.3389/fpsyt.2021.671442

**Published:** 2021-06-23

**Authors:** Michelle Curtin, Jennifer Downs, Amber Hunt, Emily R. Coleman, Brett A. Enneking, Rebecca McNally Keehn

**Affiliations:** ^1^Division of Child Development, Department of Pediatrics, Riley Child Development Center, Indiana University School of Medicine, Indianapolis, IN, United States; ^2^Division of Child and Adolescent Psychiatry, Department of Psychiatry, Indiana University School of Medicine, Indianapolis, IN, United States; ^3^Indiana University School of Medicine, Indianapolis, IN, United States

**Keywords:** medical education, adolescent, primary care, depression, suicidality, underserved

## Abstract

**Background:** Internationally, pediatric depression and suicide are significant issues. Additionally, in the context of the COVID-19 pandemic, pediatric mental health needs are rising astronomically. In light of Child & Adolescent Psychiatrist (CAP) subspecialist shortages in the United States (US), there is an increasing call for primary care physicians in Family Medicine and Pediatrics to address an increasingly broad variety of patient needs. Here we report on the development and preliminary evaluation of medical student and resident perceptions on the “INteractive Virtual Expert-led Skills Training” (INVEST) medical education curriculum, a virtual synchronous CAP curriculum employing active learning strategies, including expert-led discussion and video modeling, and discussion designed to meet those priorities.

**Methods:** In a standardized 60-min training format, our curriculum leverages audience response system polling, video modeling of key clinical skills, and interactive discussion with an expert subspecialist, over a virtual video conferencing platform. The primary educational strategy relies on use of video modeling to demonstrate best practice with CAP led group discussion to solidify and explain important concepts. Five waves of medical students and residents (N = 149) participated in the INVEST curriculum and completed pre- and post-training surveys regarding knowledge and comfort in the management of pediatric patients with depression and suicidality.

**Results:** Trainee participants reported significant positive gains in perceived likelihood of encountering pediatric suicidality as well as knowledge/comfort with depression screening and suicidality assessment in a primary care setting. Across some competency areas, there was an effect of medical learner level. Learners at lower levels generally reported the highest benefit. Medical students reported significant increases in their comfort interpreting and discussing positive depression screens and evidenced the greatest relative benefit in comfort with discussing suicidality.

**Conclusion:** To our knowledge, INVEST is the first fully virtual, multimodal curriculum led by expert CAP subspecialists. Our findings suggest that INVEST shows promise for equipping medical learners with baseline knowledge for caring for patients with pediatric depression and suicidality. This synchronous, virtually delivered curriculum allows for critical training delivered to diverse medical learners regardless of geographic location, a particular benefit during the current COVID-19 pandemic.

## Introduction

Internationally, pediatric depression and suicide are significant issues. Depression impacts ~6.2% of children and adolescents ages 5–17 years (1), resulting in impact to school performance and relationships. Compared to adults, diagnosis and treatment for children and adolescents are much more challenging (2) and subsequently only half of adolescents with depression are diagnosed before adulthood (3). While for individuals 15–29 years of age it is well documented that suicide is the second leading cause of death (4), information on suicide in pre-adolescents is less readily known; one study estimated the mean rate of suicide in children and young adolescents up to 14 years is ~0.6/100.00 worldwide (5). Additionally, in the context of the COVID-19 pandemic, pediatric mental health needs are rising astronomically (6). With the staggering impact of depression and suicidality on children and teens, identification and treatment are critical. The need now, more than ever, for virtual trainings (7) which target these skill areas, allowing access to real-world situations in low risk environments (8, 9) across diverse geographic locations (10) has become urgent.

However, severe workforce shortages exist in the Child & Adolescent Psychiatry (CAP) workforce (11, 12), which impact patient access to quality care (13–16) particularly in rural areas (11, 15). In light of CAP subspecialist shortages in the United States (US), there is an increasing call for primary care physicians in Family Medicine and Pediatrics to address an increasingly broad variety of patient needs, including mental health conditions such as depression and suicidality (14, 17, 18). However, primary care providers (18, 19) and residents in primary care residency training programs (17, 20, 21) report inadequate knowledge and skill in the assessment, diagnosis and treatment of these areas. To care for these patient needs, medical providers must acquire basic competence in pediatric mental and behavioral health (14, 22), including development of both interpersonal communication skills (23) and content knowledge (24–26).

Targeted curricula that employ novel training modalities (27–30) that make use of the available tools (31–33) are needed to make optimal use of the limited CAP workforce available as teaching experts (12, 13, 15, 16, 34). The “Guidelines for Adolescent Depression in Primary Care” for instance address steps to practice transformation to initiate identification and initial management of adolescents with depression in the primary care setting (31). In the United States, the American Academy of Child & Adolescent Psychiatry has proposed “Best Principles for Integration of Child Psychiatry into the Pediatric Health Home” focused on principles to apply the medical home model to psychiatric care needs for this population (35). Other tools, such as “Project TEACH's free library” of mental health screen “Resources”^1^, the American Academy of Pediatrics “Mental Health Toolkit” ^2^, and the PHQ-9 depression screen, are additionally being shared virtually to support practice expansion to care for the mental health needs of children and adolescents. While all of these guidelines and tools offer practices the ability to self-guide with evidence-based information around pediatric depression and suicidality, there is still a role for interactive training to build competency for providers.

To address the need for new ways of supporting primary care skill development to address the soaring pediatric depression and suicidality rates (1, 4, 5) in light of the international CAP workforce limitations (11, 12, 36), our team sought to develop a virtually-delivered medical education curriculum on the care of these patients. This virtual training was meant to suit a traditional 60-min didactic teaching block for medical students and residents in the primary care field of Family Medicine. The team's goal was to improve the awareness and comfort in responding to positive screening results of pediatric depression and suicidality in the outpatient setting of participants. Here we report on the development and preliminary evaluation of medical student and resident perceptions on the “INteractive Virtual Expert-led Skills Training” (INVEST) medical education curriculum (here after referred to as our curriculum), a virtual synchronous CAP curriculum employing active learning strategies (20, 37, 38) of expert-led video modeling, and discussion designed to meet those priorities (39).

## Methods

This study was determined to be “Protocol Exempt” following review by Indiana University IRB (approved 2/17/2020; reference number 2001953926A001). All methods were performed in accordance with the relevant guidelines and regulations. Information on the voluntary nature of the research study for informed consent was provided on all pre- and post-surveys.

### Development of INVEST Curriculum

Developed by a multidisciplinary team of subspecialists at Indiana University School of Medicine (IUSM), our curriculum was designed as a fully virtual multimodal training curriculum (8, 17, 19, 22, 24, 30, 40, 41) delivered by CAP faculty members. Multimodal trainings (20, 22, 42) use a variety of curriculum delivery methods in a single training (e.g. video modeling, readings, didactic sessions, expert feedback, etc.) and have demonstrated longer lasting impact on skill development compared to traditional, lecture based education (42–45) with improved patient outcomes (44, 46), enhanced clinician skills in discussion of sensitive topics (9, 47–49), enhanced engagement with vulnerable patients (20, 50, 51), and greater facilitation of linkages with community support resources (9). Our curriculum format was grounded in adult learning theory to address the varied experiences in the clinic and classroom of medical students and residents, building new information over their existing scaffold, and supporting lifelong learning tendencies (19, 52, 53). Ongoing financial and labor investment (38) were minimized through the development of a single structure that could be given by multiple personnel in the field without significant curriculum specific training. The curriculum structure is designed to promote active learning in primary care students and residents through particular focus on the (1) impact of mental and behavioral health condition(s), (2) available screening tool(s), and (3) patient interviewing and feedback skills for use in a primary care outpatient clinical setting. Our curriculum provides educational content (20, 50) on (1) pediatric depression, including prevalence, symptoms, and key differences between pediatric and adult presentation, and (2) suicidal behavior, including features of suicidal ideation, differences between ideation and attempt, risk factors, and strategies for discussing suicidality with patients (Supplementary Material).

**Figure 1:**
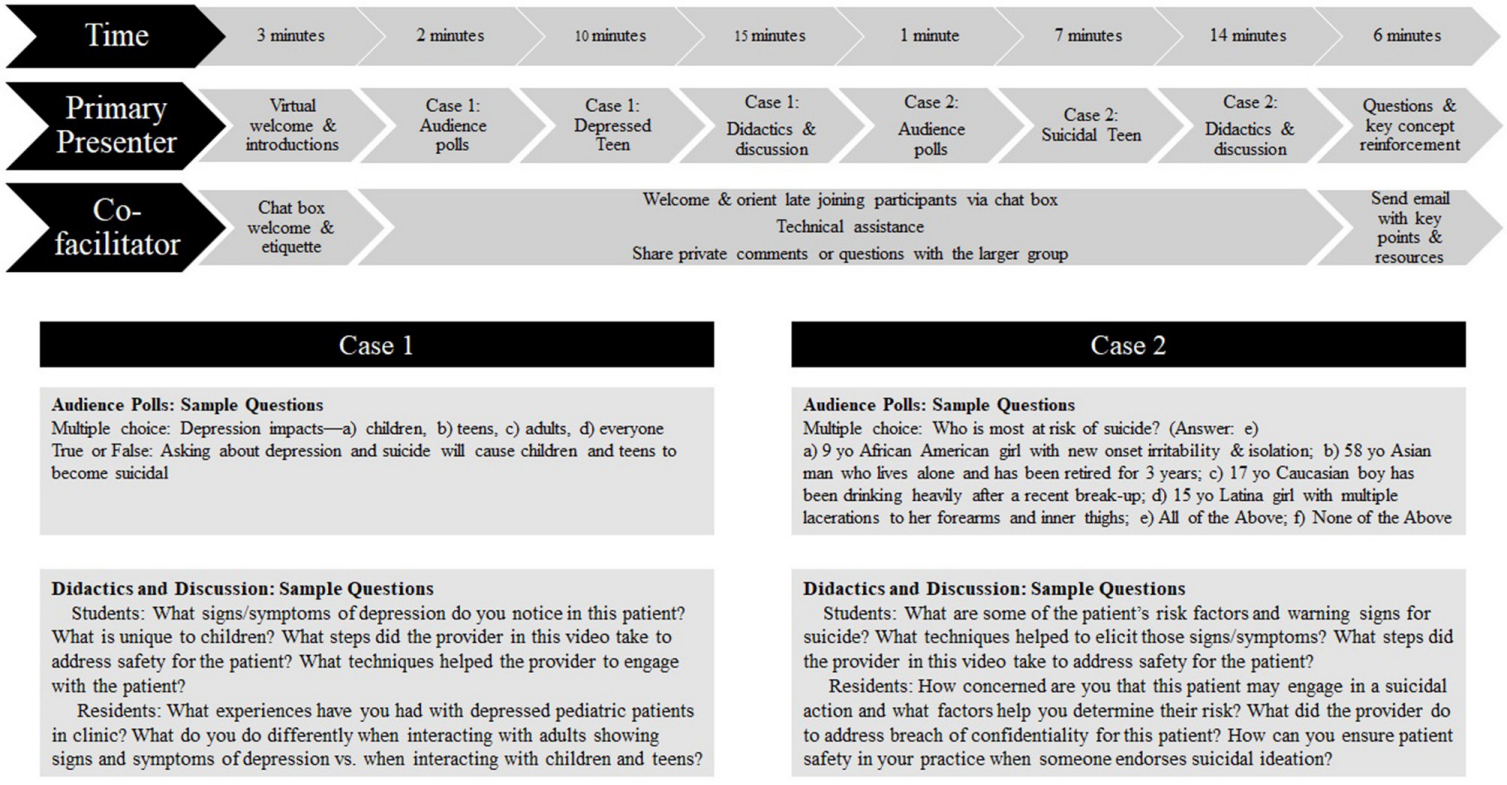
INVEST Curriculum. Content above delivered by faculty boarded in Child & Adolescent Psychiatry. Supplemental work was performed by the co-facilitator, including chat box technical assistance, trainee engagement, and collecting trainee questions and concerns.

In a standardized 60-min training format, our curriculum leverages audience response system polling, video modeling of key clinical skills, and interactive discussion with an expert subspecialist, over a virtual video conferencing platform (see Figure 1). The primary educational strategy relies on use of video modeling to demonstrate best practice with CAP led group discussion to solidify and explain important concepts. Our team produced two recorded video models of outpatient pediatric primary care appointments with a patient actor that demonstrated behavioral health assessment competencies as well as coordination and communication between the physician, patient, and office support staff. The first 10-min video models a clinician interviewing an adolescent patient with a positive Patient Health Questionnaire-9 (PHQ-9)^3^, which is widely-used in primary care for depression screening, augmented by discussion on the PHQ-9 results and interpretation, key history taking for depression diagnosis, and discussion of symptoms unique to pediatric patients. The second 7-min video models a clinician addressing a positive PHQ-9 with endorsement of suicidality symptoms, including performing a safety assessment and consideration of how to discuss need for disclosure to the adolescent's caregivers. These video modeling examples lay the groundwork for interactive discussion between learners and the CAP subspecialist, used in conjunction with virtual polling to address common misperceptions around pediatric depression and suicidality, and a limited number of content slides to ensure all attendees received an overview of pediatric depression and suicidality.

Our curriculum was initially delivered to 30 medical students and residents; pre- and post-training surveys were collected (see survey description below) and qualitatively used to improve the curriculum. Adaptations made following this trial included: (1) addition of a co-facilitator to provide real-time technical assistance, promote discussion via virtual platform chat box, and facilitate and manage discussion flow, (2) creation of discussion prompts (tailored for student or resident learners), (3) use of a mid-point discussion break in the first 10 min video to promote additional discussion, and (4) implementation of a post-training follow-up email with key curriculum points and resources (see Figure 1 for a flow chart of INVEST).

The final curriculum included a primary presenter and a co-facilitator who provided education as a team. While the topics of pediatric depression and suicide were consistently taught via videos, polling questions, content slides, and discussion questions (set at level for either residents or medical students), each time the training was given some variability occurred during discussion based on questions and/or comments from the participants. Secondary to a total 60-min available for the training, each of these components was time limited. The primary presenter focused on delivery of content including running the polling software, facilitating group discussion around the video models, and delivering content (e.g., key points). The co-facilitator provided technological support throughout the presentation by helping participants who logged on after the presentation had started, collecting chat box questions (public and private) which the primary presenter could not see in presentation mode, supporting group engagement via chat box prompts, and anticipating or responding to technical issues. A follow up email with a content summary and clinical resources was sent to all attendees following the close of the presentation.

### Assessment of INVEST Curriculum

Following Cook and colleagues' (39) framework for medical education research, a modified justification study design was used to assess learner perceptions about curriculum impact. Although justification designs often compare one educational intervention to another, a first step in assessing intervention efficacy is often to examine pre-post changes across a number of different learning domains (e.g., learner knowledge/attitude, clinical diagnosis, clinical interventions, teaching and leadership, assessment). In the present study, we focus on evaluating learner changes in knowledge and attitudes following engagement in the training. In order to measure this outcome, a self-report survey was developed to assess change across key learning objectives of the our curriculum. The survey consisted of seven items scored on a five-point Likert scale (see **Table 2** for survey items). Likert scale anchors for survey items are as follows: Items 1/2: 1 (Very unlikely/rarely) - 5 (Very likely/at least weekly); Items 3/6: 1 (Nothing) - 5 (A great deal/I could explain it to others); Items 4/5/7: 1 (Very uncomfortable/I'd try to avoid doing it) - 5 (Very comfortable/would not hesitate).

### Participants

Our curriculum was delivered to medical students and Family Medicine (FM) residents across two institutions in five waves from 5/1/2020 to 6/30/2020. In all trainings, the presenters were generally unknown to participants. Curriculum participants were recruited through (1) medical student special interest group events (Pediatrics, Neurology, Psychiatry) which were open to any student, and (2) FM program directors seeking opportunities for resident didactic education in pediatrics and mental and behavioral health. In total, 170 participants completed our curriculum; 149 participants completed pre- and post-surveys (response rate = 88%). See Table 1 for participant characteristics.

**Table 1:** Participant characteristics: graduate degree, medical training level, and medical specialty area of the participants (*N* = 149).

**Participants**	***n* (%)**
**Graduate degree**	
MD	70 (47.0)
DO	79 (53.0)
**Medical trainee level**	
MS I	10 (6.7)
MS II	20 (13.4)
MS III	29 (19.5)
MS IV	49 (32.9)
PGY I	19 (12.8)
PGY II	10 (6.7)
PGY III	11 (7.4)
PGY V	1 (0.7)
**MS intended specialty^a^**	
Pediatrics	37 (34.3)
Family medicine	30 (27.8)
Other	19 (17.6)
Undecided	8 (7.4)
Psychiatry	7 (6.5)
Pediatrics-based combo residency	4 (3.7)
Neurology/child neurology	3 (2.8)
**Resident specialty^a^**	
Family medicine	38 (95.0)
Neurology/child neurology	1 (2.5)
Other	1 (2.5)

a *Primary care: pediatrics, family medicine, internal medicine, internal medicine-pediatrics; Specialty care: psychiatry, neurology, OB/GYN, emergency medicine, dermatology, surgery, orthopedics, sleep medicine*.

### Data Collection and Analysis

Pre- and post-training surveys were collected electronically via REDcap (54, 55). Non-parametric statistical analyses were completed using SPSS Statistics 27.0 (56). We explored differences from pre- to post-training for all participants using Wilcoxan Signed Rank tests. To examine the effect of medical trainee level, participants were grouped based on clinical skill development: MS I-II (*n* = 30); MS III (*n* = 29); MS IV (*n* = 49); PGY I (*n* = 19); PGY II-III (*n* = 21). To explore effects of medical student intended specialty, two groups were created: (1) primary care (*n* = 36), and (2) specialty care (*n* = 15); see Table 1 for description of specialty grouping [Insert Table 1]. Using Kruskal-Wallis tests, we examined the effects of medical trainee level and intended medical specialty on change in scores from pre- to post-training by creating difference scores for each survey item. When a significant main effect was present, we conducted *post hoc* analyses using Mann-Whitney tests and Wilcoxan Signed Rank tests.

## Results

See Table 2 for item-level descriptive statistics for both pre- and post-scores for all participants, and participants by medical trainee level (student or resident year) and medical specialty. See Table 3 for item-level descriptive statistics for difference scores by training level and medical specialty.

**Table 2:** Item-level survey descriptive statistics for all participants (*N* = 149) by level and medical specialty area.

**Survey Item**	**Median (Range)**															
	**All Sample**		**MS I-II**		**MS III**		**MS IV**		**PGY I**		**PGY II-III**		**MS-IPC**		**MS-ISC**	
	**Pre**	**Post**	**Pre**	**Post**	**Pre**	**Post**	**Pre**	**Post**	**Pre**	**Post**	**Pre**	**Post**	**Pre**	**Post**	**Pre**	**Post**
(1) How likely do you think you are to encounter child/adolescent patients with depression?	4 (4)	4 (4)	4 (4)	5 (4)	4 (4)	5 (3)	4 (4)	4 (4)	4 (3)	3 (3)	4 (3)	4 (2)	4 (4)	5 (3)	4 (4)	4 (4)
(2) How likely do you think you are to encounter child/adolescent patients with suicidal thoughts/feelings/behaviors?	3 (4)	4 (4)	3 (4)	4 (4)	3 (4)	4 (4)	3 (4)	4 (4)	3 (3)	3 (3)	3 (4)	3 (3)	4 (4)	4 (3)	3 (3)	3 (4)
(3) How much do you know about depression in children/ adolescents?	3 (4)	4 (4)	2 (2)	3 (4)	3 (4)	4 (2)	3 (2)	4 (2)	3 (2)	3 (3)	3 (2)	4 (2)	3 (4)	4 (3)	2 (2)	3 (3)
(4) How comfortable do you feel interpreting a depression screener?	3 (4)	4 (4)	3 (3)	3 (4)	3 (2)	4 (3)	3 (3)	4 (2)	4 (3)	4 (3)	4 (2)	4 (2)	3 (3)	4 (4)	3 (3)	4 (3)
(5) How comfortable do you feel discussing a positive screen?	3 (4)	4 (3)	2 (3)	4 (3)	3 (4)	4 (3)	3 (3)	4 (2)	4 (4)	4 (2)	4 (2)	4 (2)	3 (4)	4 (2)	3 (3)	3 (3)
(6) How much do you know about performing a suicidality assessment?	3 (4)	3 (4)	2 (4)	3 (4)	3 (3)	3 (3)	3 (3)	4 (3)	2 (3)	3 (2)	3 (2)	4 (2)	2 (3)	3 (4)	2 (4)	3 (4)
(7) How comfortable do you feel discussing thoughts/feelings around suicidal thoughts/feelings/behaviors in children/adolescents?	3 (4)	4 (3)	2 (3)	3 (3)	3 (3)	4 (3)	3 (3)	4 (3)	3 (3)	4 (2)	4 (3)	4 (3)	3 (4)	4 (3)	3 (3)	4 (3)

*MS-IPC, medical student-intended primary care; MS-ISC, medical student-intended specialty care*.

**Table 3:** Item-level descriptive statistics for difference scores by participant level and intended medical specialty area (*N* = 149).

**Survey Item**	**Median (Range)**						
	**MS I-II**	**MS III**	**MS IV**	**PGY I**	**PGY II-III**	**MS-IPC**	**MS-ISC**
(1) How likely do you think you are to encounter child/adolescent patients with depression?	0.00 (4)	0.00 (4)	0.00 (4)	0.00 (3)	0.00 (3)	0.50 (4)	0.00 (4)
(2) How likely do you think you are to encounter child/adolescent patients with suicidal thoughts/feelings/behaviors?	0.00 (3)	0.00 (4)	0.00 (3)	0.00 (2)	0.00 (4)	0.50 (4)	0.00 (4)
(3) How much do you know about depression in children/adolescents?	1.00 (3)	1.00 (3)	1.00 (3)	1.00 (2)	1.00 (2)	1.00 (2)	1.00 (3)
(4) How comfortable do you feel interpreting a depression screener?	1.00 (5)	1.00 (2)	1.00 (4)	0.00 (3)	0.00 (3)	1.50 (2)	1.00 (5)
(5) How comfortable do you feel discussing a positive screen?	1.00 (4)	1.00 (4)	0.00 (3)	0.00 (3)	0.00 (2)	0.50 (2)	1.00 (5)
(6) How much do you know about performing a suicidality assessment?	1.00 (4)	1.00 (2)	1.00 (3)	1.00 (4)	1.00 (3)	1.00 (2)	1.00 (4)
(7) How comfortable do you feel discussing thoughts/feelings around suicidal thoughts/feelings/behaviors in children/adolescents?	1.00 (4)	1.00 (3)	1.00 (3)	1.00 (3)	0.00 (3)	1.00 (2)	1.00 (3)

*MS-IPC, medical student-intended primary care; MS-ISC, medical student-intended specialty care*.

### Item 1: How Likely Do You Think You Are to Encounter Child/Adolescent Patients With Depression?

There was no significant difference pre- to post- training in the perceived likelihood of encountering children/adolescents with depression (Z = 815.00, *p* = 0.07). There was a significant effect of medical trainee level on difference scores [H (4) = 17.74, *p* = 0.001]. Pairwise comparisons revealed that MS I-II showed significantly greater difference scores than all other training levels (all *p* < 0.046); no other pairwise comparisons across training levels were significant (all *p* > 0.16). A significant difference was found from pre- to post-training for MS I-II (Z = 114.00; *p* = 0.001) such that MS I-II participants rated perceived frequency higher following the training. This finding was not significant for any other training level (all *p* > 0.11).

### Item 2: How Likely Do You Think You Are to Encounter Child/Adolescent Patients With Suicidal Thoughts/Feelings/Behaviors?

There was a significant difference pre- to post- training in participants' perceived likelihood of encountering children/adolescents with suicidal thoughts/feelings/behaviors (Z = 1652.00, *p* = < 0.001) indicating that participants rated perceived frequency higher following delivery of our curriculum. There was no significant effect of training level [H (4) = 2.07, *p* = 0.72] on difference scores.

### Item 3: How Much Do You Know About Depression in Children/Adolescents?

There was a significant difference pre- to post- training in perceived knowledge about depression in children/adolescents (Z = 1,652.00, *p* = < 0.001) with participants rating perceived knowledge higher following the training. There was no significant effect of medical trainee level [H (4) = 2.16, *p* = 0.71] on difference scores.

### Item 4: How comfortable Do You Feel Interpreting a Depression Screener?

There was a significant difference pre- to post- training in participants' comfort interpreting a depression screener (Z = 4,379.50, *p* = < 0.001) with comfort increasing following participation in our curriculum. There was a significant effect of medical trainee level on difference scores [H (4) = 12.61, *p* = 0.01]. Pairwise comparisons revealed significant variation in difference scores between MS I-III and PGY I-III as well as between MS IV and PGY II-III (all *p* < 0.04) with lower medical trainee levels showing greater gains in comfort than higher training levels. No other pairwise comparisons were significant (all *p* > 0.10). A significant difference was found from pre- to post- training for MS I-II (Z = 238.00; *p* = 0.002), MS III (Z = 231.00; *p* < 0.001), and MS IV (Z = 572.00; *p* < 0.001), such that participants across these medical trainee levels rated their comfort higher following participation in our curriculum. This finding was not significant for PGYI-III (all *p* >0.08).

### Item 5: How Comfortable Do You Feel Discussing a Positive Screen?

There was a significant difference pre- to post- training in participants' comfort discussing a positive depression screener with patients (Z = 2,854.00, *p* = < 0.001) with comfort increasing following the training. Additionally, there was a significant effect of medical trainee level on difference scores [H (4) = 16.00, *p* = 0.003). Pairwise comparisons revealed significant variation in difference scores between MS I-IV and PGY II-III as well as between MS I-II and PGY I (all *p* < 0.03) with lower medical trainee levels showing greater gains in comfort than higher medical trainee levels. No other pairwise comparisons were significant (all *p* > 0.10). A significant difference was found from pre- to post- training for MS I-II (Z = 204.00; *p* < 0.001), MS III (Z = 121.00; *p* = 0.003), and MS IV (Z = 314.50; *p* < 0.001), with these participants rating their comfort higher following participation in our curriculum. This finding was not significant for PGY I-III (all *p* > 0.25).

### Item 6: How Much Do You Know About Performing a Suicidality Assessment?

There was a significant difference pre- to post- training in participants' perceived knowledge about performing a suicidality assessment (Z = 6,186.00, *p* = < 0.001) with participants reporting increased knowledge following participation in our curriculum. There was no significant effect of medical trainee level [H (4) = 1.22, *p* = 0.88) [H (1) = 0.003, *p* = 0.96) on difference scores.

### Item 7: How Comfortable Do You Feel Discussing Thoughts/Feelings Around Suicidal Thoughts/Feelings/Behaviors in Children/Adolescents?

There was a significant difference pre- to post- training in participants' comfort discussing suicidal thoughts/feelings/behaviors in children/adolescents (Z = 3,940.00, *p* = < 0.001) with comfort increasing following participation in our curriculum. A significant effect of medical trainee level on difference scores [H (4) = 9.57, *p* = 0.048) was found. Pairwise comparisons revealed that MS I-II showed greater gains in comfort as compared to all other levels (all *p* < 0.05); no other pairwise comparisons were significant (all *p* > 0.33). However, a significant difference was found from pre- to post- training for MS I-II (Z = 268.50; *p* < 0.001), MS III (Z = 136.00; *p* = 0.002), MS IV (Z = 394.00; *p* < 0.001), PGY I (Z = 66.00; *p* = 0.02), and PGY I-III (Z = 56.00; *p* = 0.03), indicating that while MS-I-II showed greater gains, all participants rated comfort higher following participation in our curriculum.

### All Items

There was no significant effect of specialty area on difference scores (all *p* > 0.06).

## Discussion

With an overall paucity internationally of CAP subspecialists (13–16, 34, 36) and the critical rates of pediatric depression and suicidality (1, 3, 4), there is a critical need for primary care clinicians to develop competency in screening and basic assessment of conditions traditionally managed by subspecialists, including mental and behavioral health disorders such as depression and suicidality (8, 14, 18, 19, 48, 57). To address this need, we developed our INVEST curriculum, a virtually-delivered, synchronous medical education program (8, 17, 19, 22, 24, 30, 40, 41) leveraging mixed technology (10) to bring subspecialty content to a diverse audience of medical students and residents. We applied our multimodal structure to focus on delivery of key skills in assessing pediatric depression and suicidality in the primary care setting (20, 50) through video modeled best practice with CAP subspecialist-led discussion. To our knowledge, this is the first curriculum that integrates multi-modal, active learning strategies (i.e., video modeling, interactive case discussion, virtual polling, subspecialty facilitation) in a fully virtual platform to deliver active education in CAP in a standard 60-min format. In creating our curriculum structure, concrete steps are demonstrated, discussed, and reviewed to address positive PHQ-9 screening results within an outpatient setting, serving as a guide for primary care learners in medical school and residency.

Outcome data collected across five waves of implementation suggests that, overall, medical students and residents experienced significant improvements in perceived knowledge and comfort with provision of care around pediatric depression and suicidality. As a result of the training, medical students reported significant improvement in awareness of pediatric depression as a clinical issue that is likely to be encountered regularly in the primary care setting. We found significant positive gains from pre- to post- training in medical students' and residents' perceived likelihood of encountering pediatric patients with suicidal ideation, knowledge about pediatric depression and suicidality, and comfort in administering and responding to depression screenings and suicidality assessment. While we failed to find differences in benefit of our curriculum across medical student intended specialty area, we did find an effect of medical learner level across a number of competency areas. Medical students and residents in lower educational levels generally reported the highest benefit. Specifically, medical students reported significant increases in their comfort interpreting and discussing positive depression screens and evidenced the greatest relative benefit in comfort with discussing suicidality. Together, these findings suggest that our curriculum may be particularly effective in preparing learners to enter clinical experiences where they may encounter patients with these behavioral health challenges (e.g., medical students before entering the outpatient setting, residents during orientation to training). While medical students reported greater gains in knowledge/comfort addressing suicidal thoughts/feelings/behaviors in pediatric patients, residents also endorsed perceived benefit. Interestingly, residents did endorse benefit from training in pediatric suicidality (items 2, 6, 7), suggesting that this may be a particular area of weakness for medical students and residents alike, consistent with findings from Fallucco et al. (17).

There are several possible reasons that more experienced learners (i.e., PGY2 or PGY3 residents) reported fewer gains from our curriculum, such as prior exposure to adult content or techniques (e.g., giving bad news) (29, 47, 49) in their clinical rotations, making depression content more familiar to them. It should be noted that in group discussion, resident level participants generally denied previous personal experience with pediatric patients showing symptoms of depression or suicidality. Additionally, evidence suggests that primary care graduates may perceive diagnosis and treatment of mental health issues as the responsibility of the subspecialist (19); as a result, they may have perceived the content as less relevant, impacting their own awareness of skills and attitudes in this area. It is possible as well that since the trainers were unknown to the residents and residents were required to attend the educational activity that there was more difficulty in building rapport in the virtual environment. Additionally, it should be noted that many residents who participated joined from their clinical service areas (e.g., hospital work room space) and may have had divided attention because of their caretaking responsibilities during the activity. It should be noted that in the cohort of medical students, a significant number of individuals were recruited via special interest groups. Therefore, the medical student audience may have been more willing to engage in the virtual training modality, as those individuals likely were already interested in the topics or population. These individuals also may have been more willing to report improvements in their understanding of the topics covered because of this interest.

Initial outcome data is promising, however there are several limitations to our approach that warrant discussion. First, outcomes were targeted to the learning objectives such that other needs/gains may have been missed. Further, outcomes were measured via participant self-report only, prohibiting study of objective change in clinical competence, and only pre- and immediate post-data was collected (total 2 data points). Collection of data at a third time point (i.e., follow-up) would strengthen the study and allow for generalization regarding maintenance of gains over time. Collection of qualitative information from participants as well as measures of engagement or related constructs from facilitators would have allowed for richer outcome data. While we did collect and analyze the effects of medical trainee level and intended specialty area, we did not collect demographic information on to minimize the burden of questionnaires for participants; this will be important in the future. Finally, further adaptation of our curriculum to teach pediatric depression and suicidality information is warranted. As all groups reported increased knowledge regarding suicidality topics, it may be beneficial to focus on this topic through development of a stand-alone module, emphasize the relationship between depression and suicidality, and/or share common content misconceptions.

### Benefits and Challenges of Virtual Delivery of Medical Education

Our curriculum provided learners with a succinct introduction to a complex topic with multi-modal and activity learning strategies. Participants additionally had the opportunity to have direct contact with CAP subspecialists, who would otherwise have been inaccessible during their medical trainee years (11, 12). While CAP subspecialist time was still required for the 60 min trainings, the overall time required was minimized by eliminating travel (+2 h to some sites) and development of a multimodal (20, 22, 42) structure (i.e., videos, polling), which allowed the CAP educator to customize conversations and discussion to the needs of the attendees while optimizing individual efforts. Using CAP subspecialists for medical training in this way may increase primary care workforce competence in subspecialty areas with low access/high need. CAP subspecialists are particularly well-equipped to address very challenging cases brought by more senior learners (PGY2 or PGY-3) to discussion, build pediatric content on the foundation.

An additional strength of our curriculum is the completely virtual delivery paired with synchronous teaching and direct engagement with CAP subspecialists (22, 37, 40). This educational format provides the opportunity for active (20, 37, 38) learning while individuals remain socially distanced, a necessity during the time of COVID-19. The virtual format also expands educational reach to diverse geographic locations, across programs or institutions, as well as across education levels or specialty areas. Though our curriculum was delivered to medical students and residents, the format can be applied to continuing medical education around subspecialty topics, including new or updated best practices for established providers.

Despite clear benefits to developing and deploying virtual medical education curricula, unique challenges exist for educators. For instance, participants may tune in from disruptive environments (e.g., while driving or from shared clinical workspace). We found that medical students and residents who shared a log-in or who joined without video were less likely to actively participate with the group and did not add comments or questions verbally or via chat box. Engagement was enhanced when participants were provided with instructions about the virtual platform prior to the training, including the directive to log on individually with camera and microphone. Additionally, the co-facilitator significantly contributed to engagement by sharing technical information, initiating, and elaborating on discussion topics, and managing participation via the virtual platform chat box.

### Future Directions

In the future, it will be important to measure changes in clinical competency following engagement in our curriculum. Other training programs have used post-training online cases with virtual patients (58) or standardized patient-based simulation (48, 59) as a means of observing clinician skill development. Chart review of referral practice, screening tool use or other skills could be another means of looking at practice changes following training and could be made into a senior level quality improvement project for practices or residents. Further, as discussed by Cook, Bordage, & Schmidt, investigation is also needed around how the multimodal format of our curriculum directly compares to other training methods addressing pediatric depression and suicidality (39). In particular, traditional didactic and more active formats like patient-based simulation should be compared, as well as objective examination of the mechanisms of learning change following engagement in the curriculum.

Future adaptations of our curriculum should target other high-need areas for primary care, including other content areas such as Autism Spectrum Disorder or Intellectual Disability or to enhance psychosocial skill training across diagnoses in the clinical environment [e.g., see Roter et al. (23) for further description of how teaching communication and psychosocial skills to address patient psychosocial distress can improve resident diagnostic accuracy and management]. Finally, our curriculum could be expanded to address practice quality improvement issues (e.g., implementing screening tools into practice) while addressing concerns about time and reimbursement [e.g., see Horwitz et al. (18)].

To improve our model, additional methods of content building, particularly for senior level residents (PGY-2 or PGY-3), and engagement must be considered. Senior level residents may benefit from additional responsibility during training, such as by bringing clinical examples, relating material to specific clinic encounters, directing learning with prepared questions, or taking the place of the co-facilitator to support discussion and technical assistance. Our curriculum could also be integrated with other forms of skill application for senior level residents, such as through facilitated role play, recorded encounters with subsequent self-rated practice (17, 20, 41), pediatric simulation (60), or unannounced standardized patients (20, 21) for formative or summative assessment (7, 28). Further, residents may be better engaged with other adaptations, such as inclusion of a familiar faculty member or a more senior resident, in order to more effectively support trainees in their exploration of knowledge gaps or feelings of vulnerability regarding difficult cases. Integrating additional tools such as skills checklists for observers to complete while viewing the video models (9, 17, 21, 27, 41) may also enhance engagement.

## Conclusions

Given the international dearth of CAP subspecialists, medical professionals will ultimately be faced with assessing and treating conditions traditionally managed by these clinicians. Thus, it is critical to develop and test effective, efficient, and financially feasible methods of training in these areas. To our knowledge, our curriculum “INVEST” is the first fully virtual, synchronous multimodal curriculum led by expert subspecialists in the CAP field which adheres to a traditional 60-min format. Our findings suggest that our curriculum shows promise for equipping medical students and residents with baseline knowledge when piloted to teach pediatric depression and suicidality. While further adaptation and evaluation is necessary, it is our hope that our curriculum framework can serve as a model for a broad array of virtual medical education content areas, with the ultimate goal of building medical students' and residents' interest and competence in primary care as well as underserved pediatric subspecialties.

^1^https://projectteachny.org/resources/

^2^https://toolkits.solutions.aap.org/mental-health/home

^3^https://www.phqscreeners.com/images/sites/g/files/g10060481/f/201412/PHQ-9_English.pdf

## Data Availability Statement

The raw data supporting the conclusions of this article will be made available by the authors, without undue reservation.

## Ethics Statement

The studies involving human participants were reviewed and approved by Indiana University IRB reference number 2001953926A001. Written informed consent for participation was not required for this study in accordance with the national legislation and the institutional requirements.

## Author Contributions

MC, JD, and AH developed and provided all trainings. MC drafted the initial manuscript and took primary responsibility for the manuscript development. EC managed all REDcap development and management and contributed to manuscript editing. BE and RM conducted data analysis and interpretation. AH, BE, and RM provided critical review and intellectual contributions to all manuscript drafts. All authors contributed to the article and approved the submitted version.

## Conflict of Interest

The authors declare that the research was conducted in the absence of any commercial or financial relationships that could be construed as a potential conflict of interest.
