# Supplementary material for: INteractive Virtual Expert-Led Skills Training: A Multi-Modal Curriculum for Medical Trainees
Source: Front Psychiatry. 2021 Jun 23;12:671442. doi: 10.3389/fpsyt.2021.671442 (PMC8260937; doi:10.3389/fpsyt.2021.671442)
Supplement: Supplementary file 1 [file Table_1.DOCX]

|  | | | | | | | | |
| --- | --- | --- | --- | --- | --- | --- | --- | --- |
| Are you a DO or MD trainee? | MD | | | | DO | | | |
| Choose your training level: | Medical student I | | Medical student II | | Medical student III | | Medical student IV | |
|  | PGY I | PGY II | | PGY III | | PGY IV | | PGY V |
| What is your current or INTENDED training? | Pediatrics | Family Medicine | | Psychiatry | | Combo (write in): | | Other (write in): |
| How likely do you think you are to encounter child/adolescent patients with depression? | 1  very unlikely/ rarely | 2  Unlikely/maybe once every 6 months | | 3  Somewhat likely/maybe once per month | | 4  Likely/maybe a few times per month | | 5  Very likely/at least weekly |
| How likely do you think you are to encounter child/adolescent patients with suicidal thoughts/feelings/behaviors? | 1  very unlikely/ rarely | 2  Unlikely/maybe once every 6 months | | 3  Somewhat likely/maybe once per month | | 4  Likely/maybe a few times per month | | 5  Very likely/at least weekly |
| How much do you know about depression in children/adolescents? | 1  Nothing | 2  A little/ I know a few basic facts | | 3  I’m comfortable with the basics | | 4  I know a fair amount beyond basics | | 5  A great deal/I could explain it to others |
| How comfortable do you feel interpreting a depression screener? | 1  Very uncomfortable /I’d try to avoid doing it | 2  Uncomfortable/  Would rather not do it, but would if I had to | | 3  Somewhat comfortable/ do it hesitantly | | 4  Comfortable/would initiate doing so when clearly needed | | 5  Very comfortable/ would not hesitate |
| How comfortable do you feel discussing a positive screen? | 1  Very uncomfortable /I’d try to avoid doing it | 2  Uncomfortable/  Would rather not do it, but would if I had to | | 3  Somewhat comfortable/ do it hesitantly | | 4  Comfortable/would initiate doing so when clearly needed | | 5  Very comfortable/ would not hesitate |
| How much do you know about performing a suicidality assessment? | 1  Nothing/ wouldn’t know where to start | 2  A little/ I know a few basic facts | | 3  I’m comfortable with the basics | | 4  I know a fair amount beyond basics | | 5  A great deal/I could explain it to others |
| How comfortable do you feel discussing thoughts/feelings around suicidal thoughts/ feelings/behaviors in children/adolescents? | 1  Very uncomfortable /I’d try to avoid doing it | 2  Uncomfortable/  Would rather not do it, but would if I had to | | 3  Somewhat comfortable/ do it hesitantly | | 4  Comfortable/would initiate doing so when clearly needed | | 5  Very comfortable/ would not hesitate |
