# Supplementary material for: INteractive Virtual Expert-Led Skills Training: A Multi-Modal Curriculum for Medical Trainees
Source: Front Psychiatry. 2021 Jun 23;12:671442. doi: 10.3389/fpsyt.2021.671442 (PMC8260937; doi:10.3389/fpsyt.2021.671442)
Supplement: Supplementary file 2 [file Data_Sheet_1.PDF]

Let’s Talk About It: Responding to a Positive Depression Screen

Michelle Curtin, DO

Jennifer Downs, MD

Amber Hunt, DO

IU SCHOOL OF MEDICINE

Welcome

- Please complete the Pre-Survey:

https://redcap.uits.iu.edu/surveys/?s=W3N8C34EFK

- Remember:
  Add your BEST email so we can share key resources and take-aways after today

POLL

Depression impacts...

children

teens

adults

everyone

Start the presentation to see live content. Still no live content? Install the app or get help at **PollEv.com/app**

DEPRESSION

- Persistent low (sad or irritable) mood with functional impairment
- 5-8% of pediatric population

VIDEO 1 (PT 1)

Discussion prompts

What signs/symptoms of depression do you notice in this patient?

What is/was unique to kids?

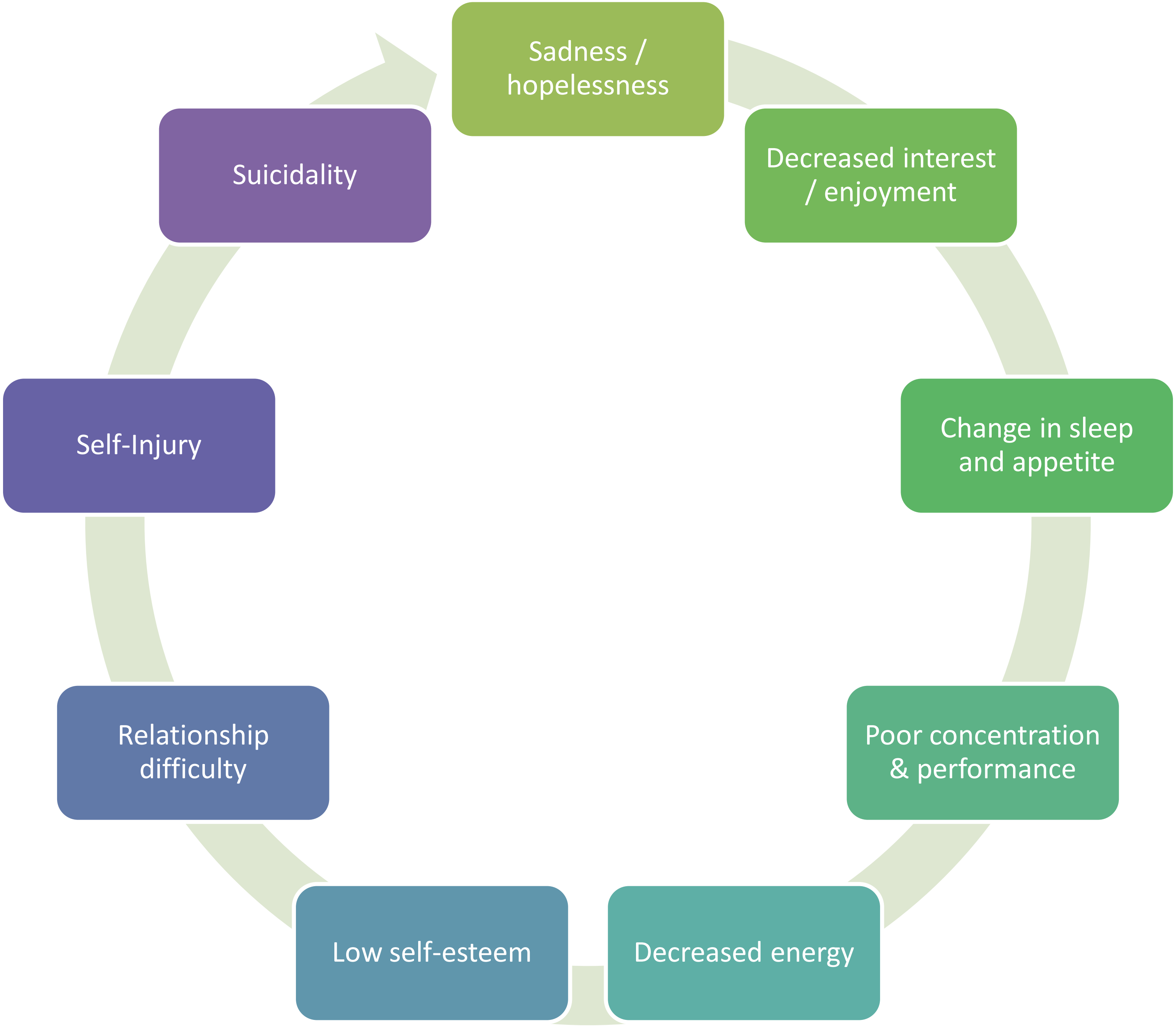
Sadness / hopelessness
Decreased interest / enjoyment
Change in sleep and appetite
Poor concentration & performance
Decreased energy
Low self-esteem
Relationship difficulty
Self-Injury
Suicidality

Kids/Teens are Different!

- Often show irritability or anger
- Display oppositional or defiant behavior
- Isolate from family and friends
- Talk about or attempt running away
- Have extreme sensitivity to rejection and failure

VIDEO 1 (PT 2)

Discussion prompts

What steps did the provider in this video take to address safety for the patient?

What techniques helped to elicit those signs/symptoms?

POLL

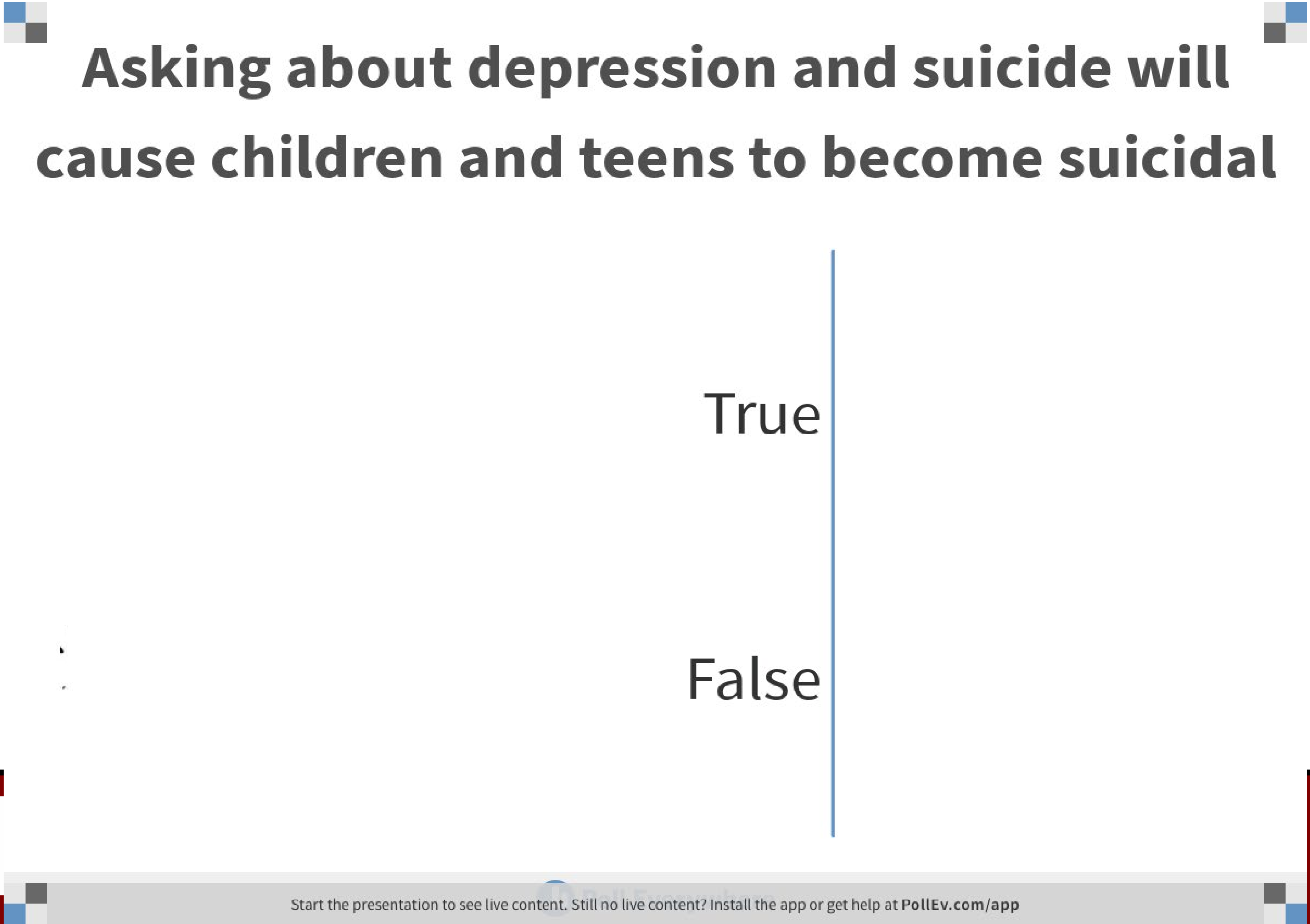
Asking about depression and suicide will cause children and teens to become suicidal
True
False
Start the presentation to see live content. Still no live content? Install the app or get help at PollEv.com/app

VIDEO 2

Discussion prompts

What are some of the patient's risk factors and warning signs for suicide?

What steps did the provider in this video take to address safety for the patient?

What techniques helped to elicit those signs/symptoms?

POLL

Which patient is likely to act on their suicidal thoughts?

9yo African American girl with new onset irritability and isolation **A**

58yo Asian man who lives alone and has been retired for 3 years **B**

17yo Caucasian boy who has been drinking heavily after a recent break-up **C**

15yo Latina girl with multiple lacerations to her forearms and inner thighs **D**

Tips when talking about suicide

- Prepare patients for breach of confidentiality
- Calmness conveys confidence
- Maintain safety at all times

Spectrum of Suicide Risk

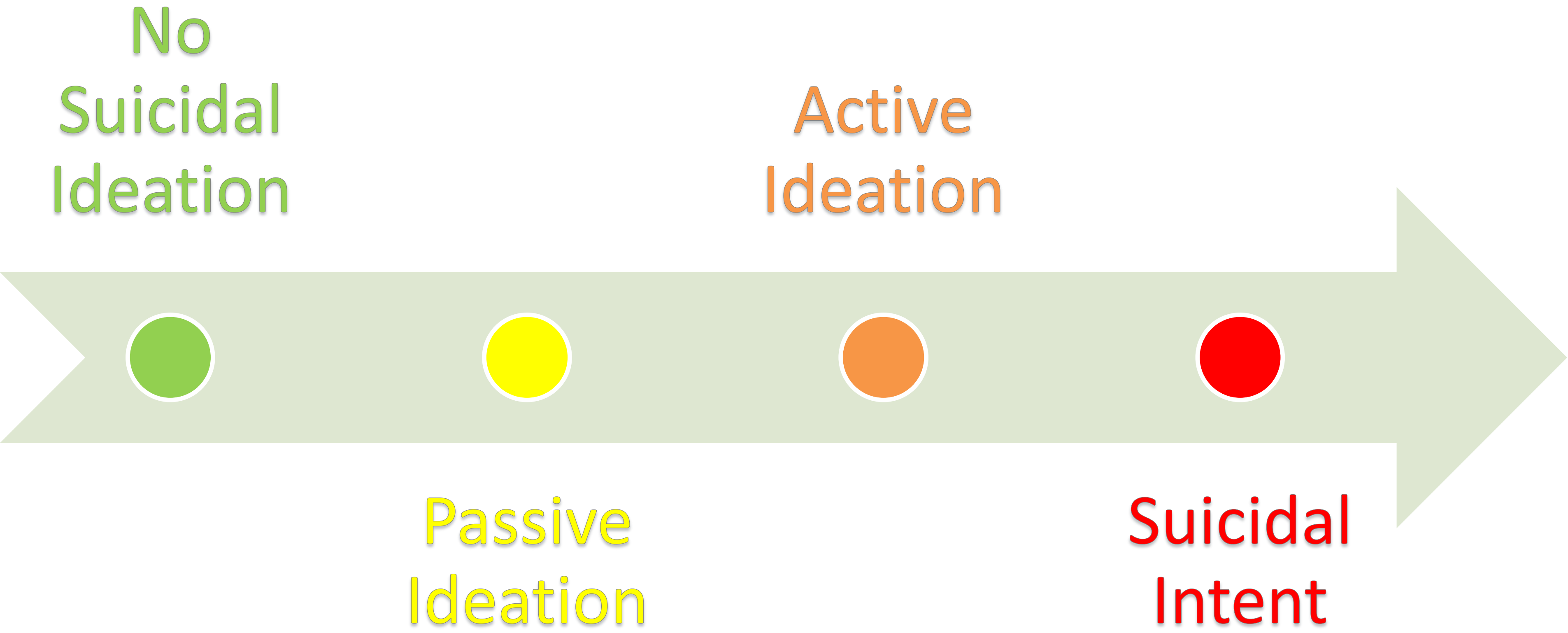

Ideation vs. Attempt

SUICIDAL IDEATION

Common

Does not equal suicide:

Need both ideation *and* lack of self preservation

Psychiatric evaluation is needed to differentiate and understand risk

SUICIDE ATTEMPT(S)

Rare & serious event

Method used is less important than patient's concept of what would happen

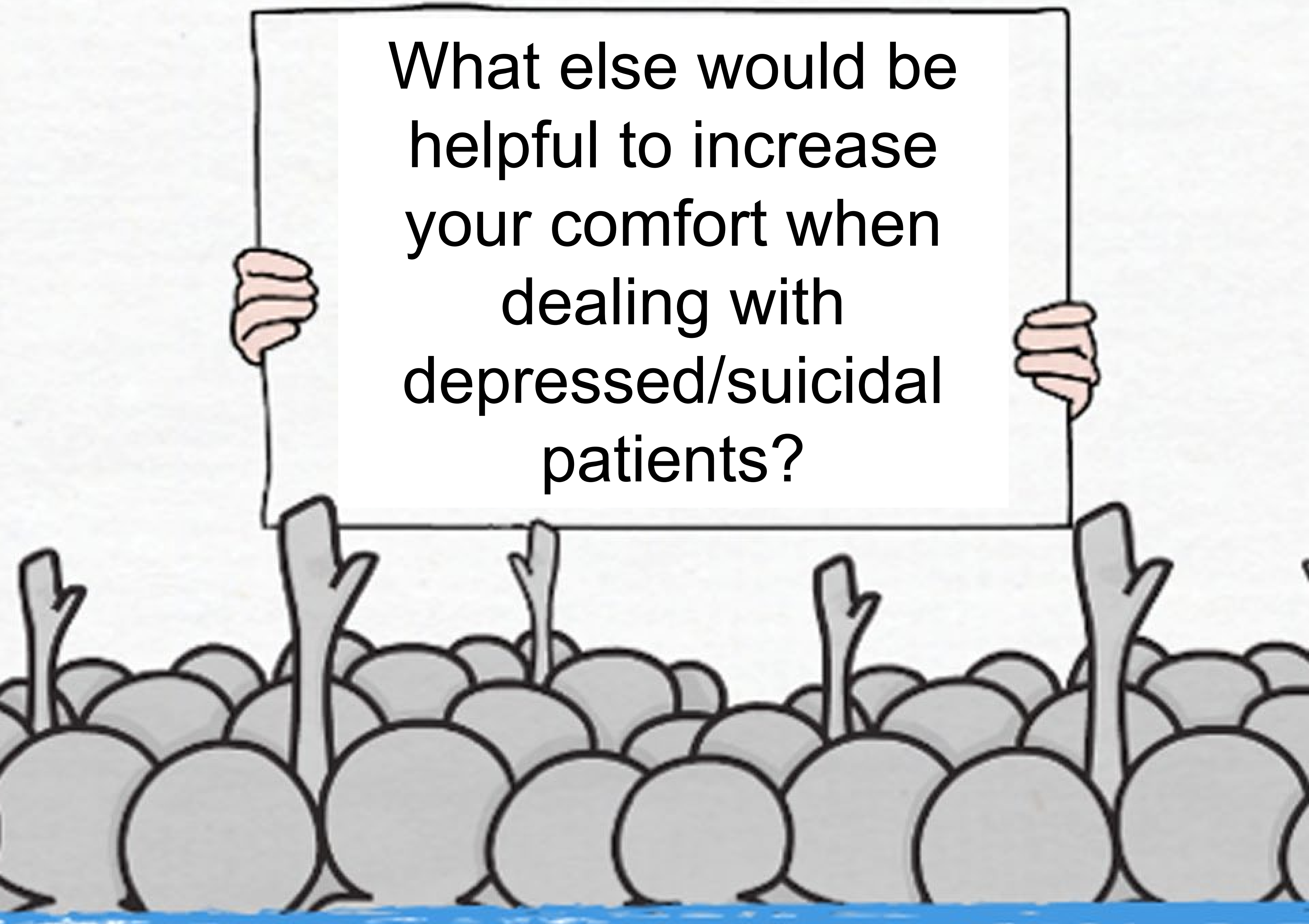
What else would be helpful to increase your comfort when dealing with depressed/suicidal patients?

TAKE AWAYS

- Depression is common
- Asking about depression/suicide does NOT cause it!
- Kids/Teens present differently
- Screening tools start a conversation
- When in doubt, reach out for help

A Post-Survey is coming to your email!
(Please complete as soon as possible.)

Thank you!

- Doctors Curtin, Downs, and Hunt

SCHOOL OF MEDICINE
